# Secretory Leukoprotease Inhibitor: A Native Antimicrobial Protein in the Innate Immune Response in a Rat Model of *S. aureus* Keratitis

**DOI:** 10.1155/2009/259393

**Published:** 2009-10-15

**Authors:** Victor E. Reviglio, Andres Grenat, Federico Pegoraro, Ruben H. Sambuelli, Tayyib Rana, Irene C. Kuo

**Affiliations:** ^1^Cornea and Anterior Segment Research, Pathology Department, School of Medicine, Catholic University of Cordoba, Cordoba, Argentina; ^2^Ophthalmology Service, Cornea Department, Cordoba Hospital, Cordoba, Argentina; ^3^Cornea and Anterior Segment Department, Northern Virginia Eye Institute, Virginia, USA; ^4^Cornea Division, Wilmer Eye Institute, Johns Hopkins University School of Medicine, Baltimore, MD, USA

## Abstract

*Purpose*. To describe the presence of secretory leukocyte protease inhibitor (SLPI), a cationic peptide with antimicrobial and antiprotease activity in the innate immune reaction in a rat model of *Staphylococcus aureus* keratitis. *Methods*. Forty female Lewis rats were divided into 2 groups: the infectious keratitis and the epithelial defect groups. Eyes were processed for immunohistochemical studies for SLPI, interleukin-1, interleukin-6, tumor necrosis factor-alpha, and matrix metalloproteinase-8. *Results*. Immunohistochemical studies confirmed high levels of SLPI, IL-1, IL-6, TNF-*α*, and MMP-8 expression in eyes with *S. aureus* keratitis and with epithelial defects, in contrast to undetectable SLPI expression in the normal control corneas. *Conclusions*. To our knowledge, this paper is the first to demonstrate the presence of SLPI with increased amounts of proinflammatory cytokines in inflamed and infected corneas.

## 1. Introduction

Infectious keratitis is a sight-threatening complication of trauma and contact lens wear [1]. *Staphylococcus aureus* can cause a virulent suppurative keratitis [2, 3]. Early diagnosis and prompt treatment are essential to allay the host inflammatory response [3]. Specific and nonspecific defense mechanisms play an important role in ocular immunity maintaining a delicate balance between effective defenses and potentially harmful inflammation responses [4]. Antimicrobial peptides contribute to innate immune defense against a number of Gram-positive and Gram-negative bacteria, viruses, and fungi [5]. These peptides include secretory leukocyte protease inhibitor (SLPI) [6–8], a cationic peptide, as well as defensins and cathelicidins.

Human SLPI is an 11.7 kDa nonglycosylated protein initially isolated from respiratory mucosal epithelial cells [8]. It is composed of two domains: a protease inhibitor at the carboxyl-terminal domain and the antimicrobial amino-terminal domain [8–10]. SLPI has defensin-like antibacterial activities and suppresses the production of inflammatory mediators [9]. In a rat model of *S*. *aureus* endophthalmitis, SLPI is present in the inflamed vitreous and retina [10]. Recent other studies demonstrate that macrophages secrete SLPI in response to proinflammatory cytokines, bacterial lipopolysaccharides, and metalloproteinases. We presume that release of SLPI by inflammatory cells in the inflamed cornea may contribute to host defense mechanisms [11].

To determine whether SLPI has a role in primary infectious keratitis, a condition in which SLPI has not been described, we investigated and quantified SLPI expression in infected and noninfected corneas using a murine model. In addition, we examined the corneal tissue for the presence of proinflammatory cytokines interleukin 1 (IL-1), interleukin 6 (IL-6), tumor necrosis factor-alpha (TNF-*α*), and matrix metalloproteinase-8 (MMP-8), as important mediators of corneal wound healing and SLPI expression.

## 2. Materials and Methods

### 2.1. Experimental Design

Animals were handled in compliance with the tenets of the Association for Research in Vision and Ophthalmology (ARVO) statement for the use of animals in Ophthalmic and Vision Research and the Guide for the Care and Use of Laboratory Animals (National Research Council). All experiments were approved by the Institutional Animal Care Committee of the Catholic University of Cordoba, Argentina.

Forty female Lewis rats, each weighing 250 g, were divided between 2 groups: the *S*. *aureus* inoculated group (20 rats) and the epithelial defect group (20 rats). The left eyes of both groups served as controls. In the *S*. *aureus* group, 10 rats were assigned to immunohistochemistry and 10 rats to Western blotting at 48 hours. The 20 rats in the epithelial defect group were divided similarly. The left control eyes were divided such that 10 eyes were assigned to immunohistochemistry and 10 eyes were assigned to Western blotting.

Wild-type *S*. *aureus* from a human endophthalmitis sample was cultured in tryptase soy broth. The bacterial suspension was centrifuged and washed with sterile saline. The suspension was serially diluted with sterile saline to 1.0 × 10^8^ CFU/mL with optical density of 0.3 at 650 nm. The *S*. *aureus* keratitis was induced based on a previous report [12].

Each rat was anesthetized with an intramuscular injection of 0.125 mL of a 1:1 mixture of 100 mg/mL ketamine and 20 mg/mL xylazine; a drop of proparacaine 0.5% was instilled in the right eye of both *S*. *aureus* and epithelial defect groups. The central 3 mm area of the corneal epithelium was scarified, followed by superficial stroma incision using a 27-gauge needle. 

The *S*. *aureus* group received 2 drops, each containing 50 uL (1.0 × 10^8^  CFU/mL) of *S*. *aureus* suspension, the epithelial defect group received 50 *μ*L of BSS, and the control group received only one drop of topical proparacaine. Biomicroscopic examination of the rats was performed 48 hours after inoculation. The rats were then euthanized using phenobarbital, and the eyes were enucleated for further study.

### 2.2. Fixation and Processing of Tissue for Immunohistochemistry

The eyes assigned to immunohistochemical study were submerged in 10% buffered formalin for 3 days, washed with distilled water, rehydrated through a graded series of ethanol, embedded in paraffin, and processed for immunohistochemistry. 

Immunohistochemical staining was performed using an avidin-biotin-peroxidase complex technique. Paraffin-embedded sections were treated with 0.6% hydrogen peroxide in methanol and blocked with 10% normal goat serum. Primary antibody consisted of 1:100 dilution of polyclonal goat anti-SLPI at 1:100 dilution (Santa Cruz Biotechnology, Inc., Santa Cruz, CA) was applied to the eye sections, incubated at room temperature for 1 hour, and the unbound antibody was removed with TBS (20 mM Tris-HCl pH 7.5, 150 mM NaCl). A biotinylated rabbit antigoat IgG secondary antibody (Vector Laboratories, Burlingame, CA) was applied and amplified with avidin-biotin-peroxidase complex (Vector Laboratories). Signals were developed for visualization with 3, 3′ diaminobenzidine. Control sections were incubated with normal goat serum. All samples were stained in parallel to minimize specimen variation. Masked pathologists graded the staining as either present or absent.

### 2.3. Western Blot Analysis

The central 4 mm zone was trephined from all right and left corneas of all groups. A 3 mm sample was then excised under a dissecting microscope by a masked pathologist and placed in a sterile tube. The tissue samples were homogenized separately in phosphate-buffered saline with 100 *μ*M butylated-hydroxytoluene and centrifuged for 10 minutes at 15 400 g. The supernatants were stored at −8°C.

The levels of SLPI, IL-1, IL-6, TNF-*α*, and MMP-8 from corneas were assessed by Western blot, with each blot being performed in duplicate. The blot was probed with purified goat polyclonal antibody from Santa Cruz Biotechnology, Inc.. For the positive control we used serum from rats with *S*. *aureus* sepsis (data not shown). Fifteen microliters of each homogenate were run under either reducing or nonreducing (r, nr) conditions at ambient temperature using a modified Laemmli method. The samples were electrophoresed on 10–15% polyacrylamide SDS gel at 100 volts for 2 hours and transferred to nitrocellulose membranes at 120 volts for 2 hours (Bio-Rad, Richmond, CA). The nitrocellulose paper was incubated at room temperature in blocking buffer (PBS, 0.05% Tween 20, 0.5% nonfat dry milk), with the primary antibody (dilution range 1:100–1:1000) and the secondary antibody (dilution range 1:1000–1:2000) for 1 hour each on a rotating platform. After three washes with TBS-T (TBS, 0.05% Tween-20), the membranes were incubated in enhanced chemilluminescence solution (Amersham Life Science, Arlington Heights, IL) followed by exposure to film. 

The values of bands from Western blots of the *S*. *aureus*, epithelial defect, and control groups were quantified using a software program for densitometric analysis (Molecular Dynamics, Sunnyvale, CA) and normalized to a standard curve to obtain relative SLPI values.

### 2.4. Statistical Analysis

Immunoreactivity was reported as the ratio of eyes positive for SLPI immunostaining to the total number of eyes. The ratio was compared to that of the control group. A *P*-value less than .05 was considered statistically significant (Fisher exact test).

The mean densitometry values from Western blots were subjected to statistical analysis to determine whether there was a difference between the *S*. *aureus* and the epithelial defect groups as compared to the control eyes. A *P*-value less than .05 was considered statistically significant (Mann-Whitney test).

## 3. Results

**Figure 1:**
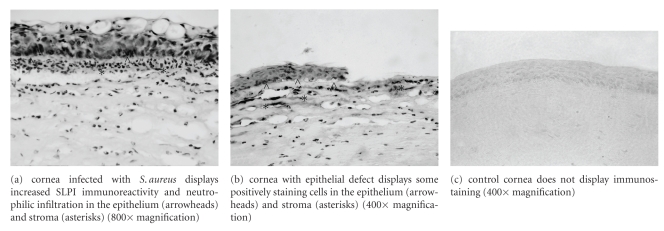
Immunolocalization of SLPI at 48 hours in corneas with *S*. *aureus* keratitis, epithelial defect, and control.

We used a murine model to help elucidate the role of SLPI, an antimicrobial peptide, in inflammatory and infectious keratitis. Forty-eight hours postinoculation, eyes from the *S*. *aureus* inoculated group demonstrated an intense inflammatory reaction at slit lamp examination. Immunohistochemical studies of the *S*. *aureus* eyes showed a neutrophilic infiltrate consistent with the inflammatory response seen on histopathologic examination. There was intense staining of SLPI localized in the eye structures compromised by inflammation; positive staining for SLPI in the corneal epithelium and stroma corroborated the slit lamp findings of keratitis (Figure 1(a)). 

Corneas in the epithelial defect group displayed defects measuring less than 1 mm at 48 hours on slit lamp examination. SLPI expression was found mainly at the levels of the corneal epithelium and anterior stroma beneath the wound, and it was associated with a weak level of neutrophilic infiltration (Figure 1(b)). The eyes from the control groups did not demonstrate clinical signs of inflammation; there was no inflammatory cell infiltrate or SLPI immunostaining of the cornea (Figure 1(c)). 

**Figure 2:**
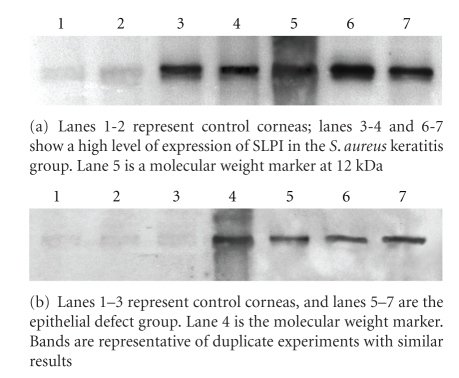
Representative Western blots of SLPI expression of supernatant samples from the *S*. *aureus*, the epithelial defect, and control groups of rat corneas.

Protein levels were measured by Western blot analysis from different groups. Similarly, immunoblots displayed a high level of SLPI expression in the *S*. *aureus* group. It was consistently observed that a lower level of expression was present in the epithelial defect group followed by the normal control group (Figure 2). 

**Figure 3:**
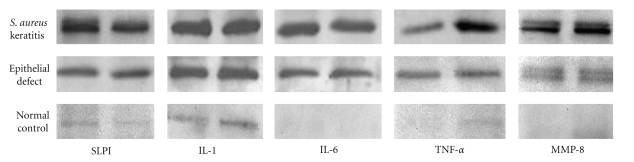
Expression of SLPI, IL-1, IL-6, TNF-*α*, and MMP-8 in *S*. *aureus*, epithelial defect, and control groups. After 48 hours, corneas were dissected and homogenized, and protein levels were examined by Western blot. Bands represent duplicate experiments with similar results.

**Figure 4:**
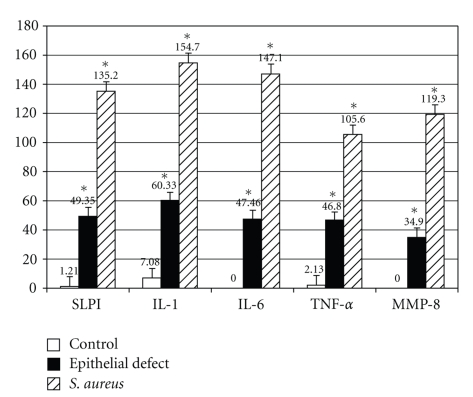
Comparison of *S*. *aureus*, epithelial defect, and control corneas in terms of quantified expression of SLPI, proinflammatory cytokines, and MMP-8 from Western blots. The results shown are the mean ± SD. Differences between values are statistically significant (*P < .05 compared with untreated control).

As we are interested in the relationship amongst SLPI expression, neutrophil recruitment, and corneal wound healing,we examined the three experimental groups for expression of proinflammatory cytokines IL-1, IL-6 and TNF-*α*, and MMP-8. Proinflammatory cytokines and MMP-8 (Figure 3) were detectable in both *S*. *aureus* and epithelial defect groups at 48 hours by Western blot technique.However, the *S*. *aureus* group showed a much higher level of expression of all proinflammatory cytokines and MMP-8 compared to eitherepithelial defect or control groups when the bands from the Western blot were quantified (Figure 4).The differences between *S*. *aureus* versus the control group and the epithelial defect group versus the control group were statistically significant (*P < .05).

## 4. Discussion

Previously, we hypothesized that SLPI plays a role in the innate immune defense of the eye in response to intraocular inflammation and infection. This study documents that SLPI is strongly expressed in a murine model of *S*. *aureus* keratitis, that SLPI expression is directly associated with infiltration by inflammatory cells in these corneas, and to our knowledge, is the first to document an association between expressions of SLPI and proinflammatory cytokines. Given what is known about the role of SLPI in other tissues such as lung, skin, and placenta [8, 13, 14] and our previous study of SLPI in a murine model of *S*. *aureus* endophthalmitis [10], our findings further support that SLPI is secreted in order to promote the early eradication of invading microorganisms and to protect the eye against proteolytic destruction by inflammatory cells. The correlation of expression of SLPI in location, time, and intensity with the primarily neutrophilic inflammatory process and with the expression of proinflammatory cytokines strongly supports that expression of SLPI is upregulated as a result of increased expression of proinflammatory cytokines.

We conclude that corneal inflammation and infection lead to SLPI expression based on the presence of elevated SLPI expression in* S*. *aureus* -infected and epithelial defect eyes, but not in normal control eyes. Furthermore, SLPI and proinflammatory cytokines were induced in the early stage of corneal wound healing in the epithelial defect group, but were expressed at lower levels compared to the levels in the *S*. *aureus* group. This suggests that neutrophils play a role in maintaining a high level of SLPI in the infected corneas. 

Corneal wound healing after injury involves a complex cascade of events involving cytokine-mediated interactions amongst the epithelial cells, activated keratocytes of the corneal stroma, components of the tear film, and cells of the immune system [12, 15]. These cytokines are multipotential proteins, playing an essential role during corneal wound healing, and they are upregulated after injury initiating the cascade of events that constitute the corneal wound healing [12, 15, 16]. Our particular interests are IL-1, IL-6, and TNF-alpha, as levels of these are significantly upregulated after corneal injury and correlated with the severity of damage [2, 3, 12, 17].

The concept that certain peptides have anti-inflammatory properties and contribute to the innate host defense has been reported in other organ systems [18]. Specifically, SLPI is involved in protection against damage from tissue inflammation [9, 12]. It also neutralizes the action of neutrophil elastase as well as other metalloproteinases secreted in the ECM [13, 19, 20]. The interactions of MMPs with specific inhibitors as well as with prostaglandins and cytokines are essential for the regulation of normal corneal wound healing [4, 15, 16, 21]. Taken together with reports that MMP-8 is present in neutrophil granules (and is also known as neutrophil collagenase), it is highly likely that neutrophils are the major source of the metalloproteinase in the current study [16]. In addition, SLPI is upregulated in response to proinflammatory cytokines and to bacterial products [9, 11]. Previous experiments show SLPI expression in bronchial, nasal, and cervical tissues, in tears, and in ocular tissue [19, 21]. Our study expands upon these findings and highlights the role of SLPI in intraocular inflammation and ocular innate immunity.

Recent studies, including a murine model of endophthalmitis, demonstrate that the SLPI is secreted by inflammatory and noninflammatory cells in response to tissue destruction [8, 9, 12, 14, 22, 23]. In conjunction with our findings, SLPI likely plays a similarly important pathophysiologic role in *S*. *aureus* keratitis. Most likely, it is expressed in response to inflammation itself, although our data do not directly rule out SLPI expression directly induced by bacterial toxins or cell wall components. In our experiments, increased expression of SLPI was found not just in association with neutrophilic infiltration, but also with increased cytokine production. As in other organ systems, expression of SLPI may be upregulated in response to proinflammatory cytokines [8, 13].

## 5. Conclusion

We demonstrate that SLPI may be secreted in order to promote the early eradication of invading microorganisms and to protect the cornea against proteolytic destruction by inflammatory cells. The known antiprotease and antimicrobial activities of SLPI suggest that its expression is actively regulated at the site of ocular tissue inflammation. In addition to supportive findings in other organ systems, our study suggests that expression of SLPI may be up regulated as a result of increased expression of IL-1, IL-6, TNF-alpha, and MMP-8. Expression of SLPI may have a role in regulation of neutrophil recruitment. Because of its endogenous antimicrobial activities and role as an inflammatory mediator, further studies addressing the role of SLPI in innate ocular immunity and in wound-healing may have consequences in the development of innovative prophylactic and therapeutic strategies for eye disease.

## References

[1] Balzli CL, McCormick CC, Caballero AR (2008). Fluoroquinolone therapy in a rabbit model of post-LASIK methicillin-resistant Staphylococcus aureus keratitis. *Journal of Cataract and Refractive Surgery*.

[2] Callegan MC, O’Callaghan J, Hill JM (1994). Pharmacokinetic considerations in the treatment of bacterial keratitis. *Clinical Pharmacokinetics*.

[3] Dajcs JJ, Thibodeaux BA, Girgis DO, O’Callaghan RJ (2002). Corneal virulence of Staphylococcus aureus in an experimental model of keratitis. *DNA and Cell Biology*.

[4] O’Callaghan RJ, Girgis DO, Dajcs JJ, Sloop GD (2003). Host defense against bacterial keratitis. *Ocular Immunology and Inflammation*.

[5] Zasloff M (1992). Antibiotic peptides as mediators of innate immunity. *Current Opinion in Immunology*.

[6] Martin E, Ganz T, Lehrer RI (1995). Defensins and other endogenous peptide antibiotics of vertebrates. *Journal of Leukocyte Biology*.

[7] Lehrer RI, Lichtenstein AK, Ganz T (1993). Defensins: antimicrobial and cytotoxic peptides of mammalian cells. *Annual Review of Immunology*.

[8] Song X-Y, Zeng L, Jin W (1999). Secretory leukocyte protease inhibitor suppresses the inflammation and joint damage of bacterial cell wall-induced arthritis. *Journal of Experimental Medicine*.

[9] Tomee JFC, Koëter GH, Hiemstra PS, Kauffman HF (1998). Secretory leukoprotease inhibitor: a native antimicrobial protein presenting a new therapeutic option?. *Thorax*.

[10] Reviglio VE, Sambuelli RH, Olmedo A (2007). Secretory leukocyte protease inhibitor is an inducible antimicrobial peptide expressed in Staphylococcus aureus endophthalmitis. *Mediators of Inflammation*.

[11] Jin F-Y, Nathan C, Radzioch D, Ding A (1997). Secretory leukocyte protease inhibitor: a macrophage product induced by and antagonistic to bacterial lipopolysaccharide. *Cell*.

[12] Girgis DO, Sloop GD, Reed JM, O’Callaghan RJ (2005). Effects of toxin production in a murine model of Staphylococcus aureus keratitis. *Investigative Ophthalmology and Visual Science*.

[13] Sallenave JM, Shulmann J, Crossley J, Jordana M, Gauldie J (1994). Regulation of secretory leukocyte proteinase inhibitor (SLPI) and elastase-specific inhibitor (ESI/elafin) in human airway epithelial cells by cytokines and neutrophilic enzymes.. *American Journal of Respiratory Cell and Molecular Biology*.

[14] Tomee JFC, Hiemstra PS, Heinzel-Wieland R, Kauffman HF (1997). Antileukoprotease: an endogenous protein in the innate mucosal defense against fungi. *Journal of Infectious Diseases*.

[15] Vaday GG, Lider O (2000). Extracellular matrix moieties, cytokines, and enzymes: dynamic effects on immune cell behavior and inflammation. *Journal of Leukocyte Biology*.

[16] Goetzl EJ, Banda MJ, Leppert D (1996). Matrix metalloproteinases in immunity. *Journal of Immunology*.

[17] Zlotnik A, Morales J, Hedrick JA (1999). Recent advances in chemokines and chemokine receptors. *Critical Reviews in Immunology*.

[18] Bals R, Wang X, Meegalla RL (1999). Mouse B-defensin 3 is an inducible antimicrobial peptide expressed in the epithelia of multiple organs. *Infection and Immunity*.

[19] Ohlsson K, Rosengren M, Stetler G, J. C.  Taylor, C. Mittman (1986). Structure, genomic organization and tissue distribution of human secretory leukocyte-protease inhibitor (SLPI): a potent inhibitor of neutrophil elastase. *Pulmonary Emphysema and Proteolysis*.

[20] Reviglio VE, Rana TS, Li QJ, Ashraf MF, Daly MK, O’Brien TP (2003). Effects of topical nonsteroidal antiinflammatory drugs on the expression of matrix metalloproteinases in the cornea. *Journal of Cataract and Refractive Surgery*.

[21] Sathe S, Sakata M, Beaton AR, Sack RA (1998). Identification, origins and the diurnal role of the principal serine protease inhibitors in human tear fluid. *Current Eye Research*.

[22] Zhang Q, Shimoya K, Moriyama A (2001). Production of secretory leukocyte protease inhibitor by human amniotic membranes and regulation of its concentration in amniotic fluid. *Molecular Human Reproduction*.

[23] Hiemstra PS, Maassen RJ, Stolk J, Heinzel-Wieland R, Steffens GJ, Dijkman JH (1996). Antibacterial activity of antileukoprotease. *Infection and Immunity*.

